# Usefulness of Microbiome for Forensic Geolocation: A Review

**DOI:** 10.3390/life11121322

**Published:** 2021-11-30

**Authors:** Christian Haarkötter, María Saiz, Xiomara Gálvez, María Isabel Medina-Lozano, Juan Carlos Álvarez, José Antonio Lorente

**Affiliations:** Laboratory of Genetic Identification, Department of Legal Medicine, Toxicology and Physical Anthropology, Faculty of Medicine, University of Granada, 18016 Granada, Spain; chaarkotter@ugr.es (C.H.); xgales@ugr.es (X.G.); miml29@ugr.es (M.I.M.-L.); juanca@ugr.es (J.C.Á.); jlorente@ugr.es (J.A.L.)

**Keywords:** forensic microbiology, forensic science, geolocation, microbiome, microbiota

## Abstract

Forensic microbiomics is a promising tool for crime investigation. Geolocation, which connects an individual to a certain place or location by microbiota, has been fairly well studied in the literature, and several applications have been found. The aim of this review is to highlight the main findings in this field, including the current sample storage, DNA extraction, sequencing and data analysis techniques that are being used, and its potential applications in human trafficking and ancient DNA studies. Second, the challenges and limitations of forensic microbiomics and geolocation are emphasised, providing recommendations for the establishment of this tool in the forensic science community.

## 1. Introduction

The microbiome is not a novel concept, given that the term was developed during the late 1980s by Whipps et al. [1] to refer to a group of microorganisms living in a defined area. The common factor uniting the various fungi and bacteria in a particular location is the location itself. Today, the term has evolved into two different concepts: ‘microbiota’ is a term used for a group of microorganisms or viruses that are centred and interact in a certain area, and ‘microbiome’ is a term for the genomic study of a community of microorganisms [2]. However, the microbiome has been defined as a certain microbial community that lives in a defined area with certain physical and chemical properties (it includes the microorganisms and their environment), and the microbiota includes an assembly of microorganisms that belong to different kingdoms, including their microbial structures, metabolic reagents or products and mobile or relic DNA/RNA elements. Thus, the original definition as stated by Whipps appears to be the most accurate [3].

**Figure 1:**
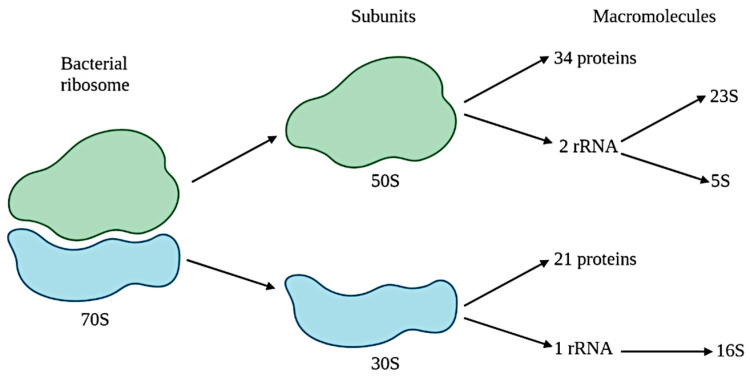
Bacterial ribosome and 16S rRNA.

Various methodologies and strategies have been developed to describe and classify microorganisms. Prior to 1960, the methodology was mostly based on morphology, metabolic requirements or pathogenicity. In 1960, a numerical taxonomy was introduced into bacterial systematics with the mol% guanine–cytosine content of DNA as a quantitative measurement. Therefore, no more than 2–3% of the variation in guanine–cytosine content was expected in the same species of microorganisms. Chemotaxonomy, the description of new species based on the study of the composition of cell walls or bacterial cytochromes, became common from 1960 to 1980; however, it was supplanted by the arrival of 16S ribosome DNA or rDNA (see Figure 1) gene sequencing during the mid-1990s. This approach implied that strains with less than 98.7% sequence similarity were a new species. Given that 16S rDNA is easily isolated, ubiquitous and constrained (constraints are mechanisms that limit or restrict adaptative evolution), it is commonly studied; that is the reason why it is the most common approach in literature [4]. Most recently, the introduction of high-throughput technologies, commonly known as next generation sequencing (NGS), allowed whole genome sequencing, in which new species are defined by the comparison between two chromosomes [5]. NGS platforms have developed various commercial kits for microbiome analysis, allowing laboratories to employ automated and software-integrated procedures.

Currently, there is no official or recognised system for the classification of bacteria; however, the most commonly used system is the polyphasic approach, which includes phenotypic, chemotaxonomic, genotypic and phylogenetic data [6]. Microbiologists use Linnaeus’s binomial naming system to designate microorganisms, with *Proteobacteria* divided into seven orders: *Chromatiales*, *Thiotrichales*, *Legionellales*, *Pseudomonadales*, *Vibrionales*, *Enterobacteriales* and *Pasteurellales*. Each order includes several genera, and each genus a variety of species; for example, the family *Enterobacteriaceae* (from the order *Enterobacteriales*) includes the genera *Enterobacter*, *Escherichia*, *Klebsiella*, *Proteus*, *Salmonella*, *Serratia*, *Shigella* and *Yersinia* [7]. 

**Figure 2:**
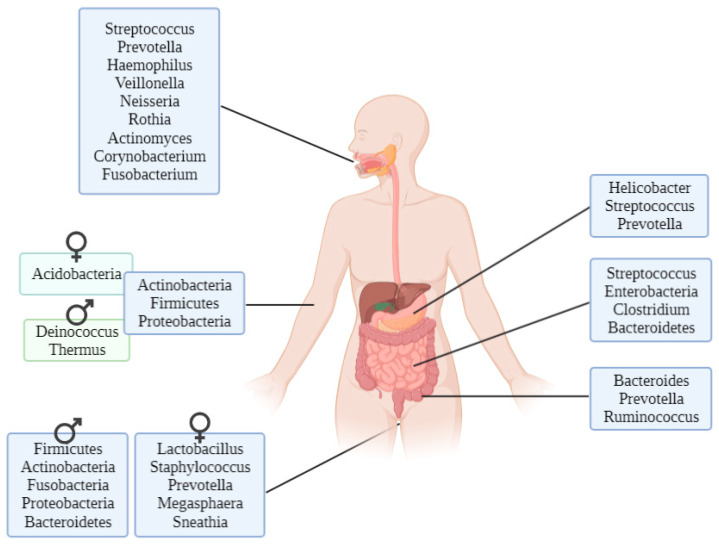
Most common bacteria genera in the different parts of the human body [9].

Given the importance of the microbiota, the Human Genome Project led to the Human Microbiome Project, whose main objectives are creating a draft database of the human-associated microbiome by 16S rRNA sequencing, studying individuals who represent specific clusters and analysing global human microbiome diversity [8]. As an example of the large variety of microbiota that live in the human body, the most common genera in stomach, small and large intestine, oral cavity, male and female urogenital system and skin are shown in Figure 2.

Forensic microbiology is a fairly new field in the forensic sciences, and it has been developing since the terrorist attacks in the United States in 2001 due to the fear of a possible biological attack. Forensic microbiologists were concerned with developing tools to identify bioweapons and those who use them [10]. Since then, and thanks to major developments in sequencing technologies, its applications are growing rapidly [11]. There are currently three main areas of interest in forensic science [12]:

*Identification*. The microbiome has the potential to identify an individual in the population based on their characteristic microbials. It appears to be possible to identify the items a person has touched, and therefore to define biogeographical patterns in the items.

*Post-Mortem Interval Estimation*. Research shows that there are distinctive microorganisms that can be sequenced at various time points and body locations during decomposition.

*Geolocation*. Microbiota differ in composition across geographical locations due to climate, rainfall, altitude, soil and energy sources in the environment; thus, the knowledge of specific bacteria composing a certain area would could link a person or item to a certain place.

In this review, we investigated state-of-the-art geolocation in forensic microbiology, exploring the main advances in recent years as well as the current challenges and limitations of this emerging tool in forensic science.

## 2. Forensic Microbiome as a Tool for Geolocation

“Every contact leaves a trace” is probably the most important axiom in forensic science, given that it was first established by Locard during the early 20th century. This statement has been applied by forensic scientists since then in all forensic fields, and it can also be applied to microbiome studies [13]. If a certain place contains a characteristic microbiota that is different from other locations, we can analyse a person’s microbiome and possibly establish where they have been, which is precisely the main principle of microbiome geolocation. 

**Figure 3:**
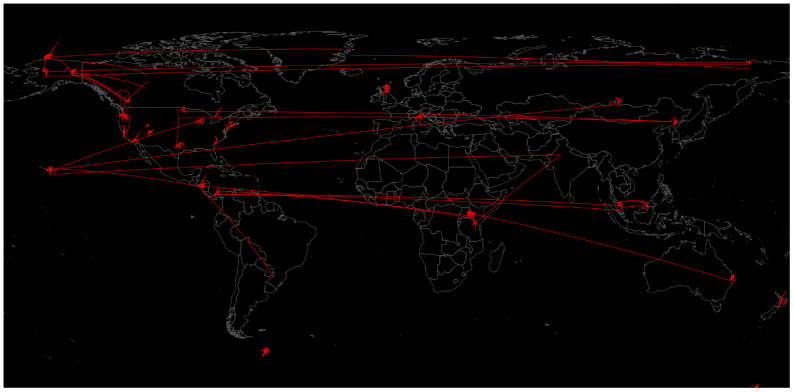
Soil microbiome samples collected by the Earth Microbiome Project by similarity [15].

Several studies have been performed to characterise the urban and transit microbiome, demonstrating that certain areas of a city contain unique microbiome profiles [14]. Along these lines, the Earth Microbiome Project, EMP (http://www.earthmicrobiome.org) must be mentioned. It was created in 2010 to sample the whole planet’s microbial communities with the aim of understanding the biogeographic variations and principles that govern microbial communities by using standardised protocols and environmental descriptors in an open science model [15]. The various samples and their connection by similarity (containing similar types of microbial communities) are shown in Figure 3.

### 2.1. Soil and Surface Microbiome

The literature has demonstrated that a whole city’s microbiome can be analysed by swab sampling of subway stations, public parks and waterways. Certain species have been found to be linked to certain areas of the city, with a degree of fluctuation observed in some genera during the day. However, an important issue was also discovered: many samples did not match any known organism [16], which calls attention to the importance of projects such as the Earth Microbiome Project. A combined effort to study the urban metagenome can be found in the Metagenomics and Metadesign of the Subways and Urban Biomes, (MetaSUB) International Consortium, which was created with the aim of helping with city planning, public health and architectural design matters [17]. Moreover, in 60 cities across a three-year longitudinal study, it was established that there is geographic variation among microbial communities in type and density [18]; thus, it is possible to create a map of the various microbiota that can be found in specific cities. Interestingly, it has been observed that a relationship can be established between a geographic metagenome and organisms’ diversity, acting as a type of ‘molecular echo’ [19]. This molecular echo could be useful information for future correlations between the microbiome and forensic entomology. Recent advances in city microbiome studies suggest that certain species are especially useful for geolocation, given that some of them are invariably present in every studied city, thus, some genera was particular to each location [20]. 

There appears to be a correlation between geographical distance and microbial communities in soil samples: the farther apart two soil samples are, the more different are their associated microbiota, differences that are non-significant at less than 2 m and that increase logarithmically in significance value from 25 m to 60 km [21]. This difference can give forensic geolocation a current error range of 25 m, which is not negligible; therefore, further studies with larger samples and regional replications should be conducted to confirm this hypothesis. Furthermore, even a mixture of soil samples can be distinguished. However, although the application of a single technique is useful for characterising a soil sample, in a study it was only possible to establish the origin of a mixed soil sample by using the combination of two different techniques: ribosomal intergenic spacer analysis (RISA) and 16S rRNA sequencing [22]. Geographically close cities display similar yet distinguishable microbiological communities; therefore, correct classification ranks can be obtained only if a strong reference database is used [23].

Although seasonal fluctuations in soil communities have been described, studies succeed when it is possible to geolocate samples despite a time lapse of several weeks, variations in temperature, rainfall, or even the desiccation of the soil sample in a car boot, which are expected scenarios when using this technique for forensic purposes [24].

Research in closed areas such as offices shows that the observed microbiome could be more a consequence of contact with the human microbiome than specific communities living in those areas; however, a small specific community can be observed on untouched surfaces. More variations were observed among floor or ceiling sampling locations than on different surface materials. Although the difference between offices is not as strong as between cities, bacterial communities present in closed areas appear to be less susceptible to temperature and humidity variations [25]. Furthermore, it appears that people and animals living in the same space share their microbial communities, probably due to direct contact. Thus, if a person leaves his or her home for several days, changes in the home microbiome are observed [26].

Altitude is another factor to be taken into account, given that skin microbiome diversity decreases with higher altitudes. This decrease has also been observed in lake water, so it can be correlated with the soil microbiome, possibly due to ultraviolet intensity, temperature or oxygen availability. A study conducted in China showed that the skin microbiome also tends to be less diverse among individuals living at high altitudes; however, each individual was distinguishable within each group, and some taxa have been observed to be more common at certain elevations [27].

### 2.2. In Vivo Microbiome

To date, soil and surface microbiome differences have been found in different geographical areas. However, it is possible to perform a geolocation on a person given their own personal microbiome. Certainly, there are variations, perhaps due to differences between factors such as the level of industrialisation, the characteristics of the geographic region or a person’s lifestyle habits [28]. 

There are genera of microorganisms that allow researchers to assess a person’s geographical origin. For example, *Helicobacter pylori* extracted from gastric mucosa has been used to determine the geographical origin of unidentified Asian cadavers, resulting in three different clusters: East Asian, Western and Southeast Asian [29]. Furthermore, studies focusing on the relationship between microbiota and diseases such as obesity have found differences between Colombians, Americans, Europeans, Japanese and South Koreans and their relative disposition to increased body mass index [30]. These differences have also been found in studies conducted to evaluate the relationship between the microbiome and infectious diseases such as *Plasmodium falciparum* infection, finding again geographical differences among people in their stool microbiota [31]. Other studies performed with human hair microbiota have found differences between samples from California and Maryland, and interestingly, scalp hair resulted in better prediction of geolocation than pubic hair [32].

*Firmicutes* and *Bacteroidetes* appear to have a certain pattern depending on the latitude. In a study conducted with healthy individuals’ gut microbiota, it was found that the *Firmicutes* and *Bacteroidetes* proportion differs with latitude: the proportion of *Firmicutes* is much higher in the Northern Hemisphere than in the Southern Hemisphere [33]. The explanation of the differences in microbiota remains unclear, although there are three proposed models: host genes, the environment itself or host plasticity.

### 2.3. Machine Learning and Geolocation

There are few research or review articles that address the cornerstone of forensic microbiome geolocation—machine learning. When a sample is processed and a read has been obtained, it still must be linked to a certain environment or area microbiome, which is only possible due to recent advancements in machine learning, including a powerful tool—random forest.

**Figure 4:**
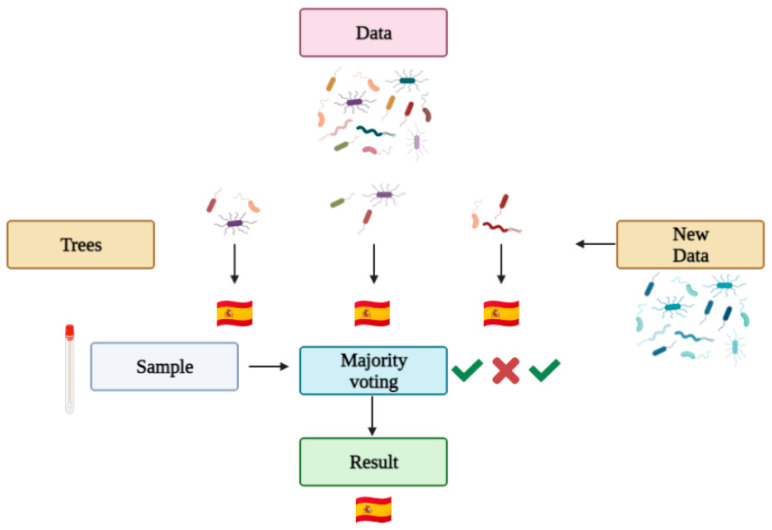
Random forest prediction in microbiome Forensics.

Machine learning automates computers to make predictions based on data. Machine learning has been used in biomedical research, cancer diagnosis and with the human microbiome to predict categorical or numerical values by classification and regression, respectively [34]. The program itself learns from each classification it makes, so the next classification contemplates the previous ones. There are numerous machine learning techniques available for the classification of the human microbiota [35], and random forest is one of them. It is the most commonly used technique in microbiome forensics [23]. A random forest algorithm is a combination of tree predictors (a tree is a type of flux diagram in which every internal node is an attribute, the branch is a decision rule and every leaf a result). Each tree has the same distribution, and its values depend on a random vector sampled independently [36]. Roughly, a random forest works as follows (see Figure 4): a data set is introduced into the algorithm, which generates the statistically best decision trees for the given variables, and the algorithm is trained so it can learn from its successes and mistakes (as any other machine learning based algorithm). Then, a problem sample is given so the algorithm makes decisions with the various trees generated, ultimately giving a category result (for example, a country) based on a majority vote of the tree results.

Although random forest is an accurate and unbiased predictor that needs no rigid statistical assumptions of the target variable, it has some disadvantages: greater computational intensity with the increase in calibration data, high sensitivity of predictions to the quality of the input data and variations in obtained model interpretation [37]. Several algorithms have been developed to make machine learning more accessible to forensic scientists. An example applied to microbiome geolocation is DeepSpace, which is based on deep neural network classifiers (algorithms of machine learning that assimilate data representation when they recognise, for example, a human face in a pixel image), which could correctly classify dust from different countries with a 90% accuracy just by using fungi data [38].

### 2.4. Protocols

Several protocols for sampling, DNA extraction and amplification are available; however, given that swabs are a reliable technique and the DNA extraction methodology is crucial, a reduction in host DNA is recommended [39].

#### 2.4.1. Sampling

The Earth Microbiome Project has designed a protocol for collaborators who want to contribute samples. The protocol depends on the specific sample type, and is summarised in Table 1.

#### 2.4.2. DNA Extraction

For good-quality environmental samples there are several commercial platforms for microbiome studies, all of which are magnetic beads based: KingFisher Flex Purification System (ThermoFisher Scientific, Waltham, MA, USA); epMotion 5075 TMX (Eppendorf, Hamburg, Germany); and Tecan Freedom EVO Nucleic Acid Purification (Tecan, Morrisville, NC, USA) [41]. The platforms have been tested with a variety of samples, including faeces, oral, skin, soil and water. The various commercial DNA extraction kits available are shown in Table 2. A special strategy has been developed for low-template microbiome samples, for as few as 50-500 cells, called KatharoSeq. It is based on Mo Bio PowerSoil and the QIAGEN Ultra Clean kit [42]. Other kits not designed for the microbiome have been validated for forensic microbiome workflows [43]; however, they present the challenge of not eliminating the non-bacterial DNA present in samples.

#### 2.4.3. Sequencing

The 16S amplification protocol was designed for prokaryotes, bacteria and archaea, given that it is an excellent phylogenetic marker, and it provides insight into both communities and individual microbial taxa. The protocol’s ability to relate trends of species to hosts or environments has been proven. The polymerase chain reaction primers were developed for the V4 region of 16S rRNA [48].

The 18S amplification protocol is designed for microbial eukaryotes with primers designed for V9 hypervariable regions of small subunit rRNA genes or for a combination of both V4 and V9 hypervariable regions [49,50].

Both amplification protocols have been validated on Illumina 454-pyrosequencing platforms. More recent devices, such as the Ion GeneStudio™ S5 System (Thermo Fisher Scientific, Waltham, MA, USA) or MiSeq FGx™ Forensic genomics System (Verogen, San Diego, CA, USA), which are being designed for forensic applications, still have to be validated for forensic microbiome workflows.

### 2.5. Current Applications

Several uses of microbiome geolocation have already been discussed, and they are summarised in Table 3. Next, we will discuss two applications based on our laboratory’s field of expertise: microbiome-related DNA solutions for human trafficking and for ancient DNA studies.

#### 2.5.1. DNA-Prokids

DNA-Prokids (http://www.dna-prokids.org) is a humanitarian and non-profit program developed by the University of Granada in direct collaboration with institutions and state organisations in various countries to fight human trafficking. The aim of the program is to create a Questioned Database using genetic data from children of unknown identity and a Reference Database with samples collected from the relatives of missing children, so they can be identified and returned to their home countries. To date, the program has collected more than 18,000 samples and identified 1800 children [52]. 

It has been proposed that microbiome data could be an interesting tool in humanitarian programs based on DNA, which is the case of DNA-Prokids [12]. In this regard, child victims of human trafficking could be tracked to their home countries, including the route they have followed from their home countries to the place where they are located, providing the police forces with very useful data to track the networks established between countries by criminal networks.

#### 2.5.2. Skeletal Remains

We have discussed the connection that can be established between the microbiome of a certain area and the person who has been in that area; however, it would also be interesting to consider the connection between the microbiota and recovered evidence and its preservation; i.e., whether microbiota correlate with a successful or failed DNA typing. Along these lines, this section focuses on skeletal remains, which are among the biological elements most exposed to a certain environment.

Microbial communities affect the body both in life (producing substances that affect bone integrity) and in death (as taphonomic agents). As the body decays, it is exposed to enteric and soil microbiomes; around the decomposing bone there is a transition from *Bacteroidetes* and *Firmicutes* to *Proteobacteria* and *Actinobacteria*, with a re-emergence of *Acidobacteria*. Local soil bacteria and fungi can cause histological alterations in bone, including linear longitudinal lamellate and budded alterations by bacteria and Wedl-type tunnelling (Wedl was a 19th-century pioneer in the microbial degradation of tooth dentine in animal bone [53]) by fungal hyphae or *Cyanobacteria* in aquatic environments. Microorganisms are believed to cause bioerosion by attacking the bone via the bone’s vascular system or by colonising the bone surface [54].

There are few studies in literature about the types of microbiota found in bones during decay. Damann et al. [55] found that the proportion of the bacteria present in bones varies with the state of decay, from partially skeletonised (*Proteobacteria*, *Firmicutes*, *Bacteroidetes*), to skeletonised (*Proteobacteria*, *Firmicutes*, *Bacteroidetes*), to dry remains (*Proteobacteria*, *Actinobacteria*, *Bacteroidetes*), whereas the soil microbiota was characterised by the presence of *Proteobacteria*, *Acidobacteria* and *Actinobacteria*. *Pseudomonadaceae* was the most common family in the first two stages of decay, whereas dry bones were characterised by *Caulobacteraceae* and other rare family bacteria. Thus, partially skeletonised samples correlated with bacteria found in the human gut, whereas dry skeletal remains tended to be more similar to soil communities [55].

Emmons et al. [51] examined the type of microbes found in each bone and their correlation to human DNA preservation. The post-mortem dominant taxa observed was the same as that found by Damann et al. with a few discrepancies possibly due to the sequencing methodology. Moreover, eukaryotic taxa such as Ascomycota and Basidiomycota were observed to be dominant, with Apicomplexa, *Ciliophora* and *Cercozoa* the most common fungi. There were differences between microbial communities by individual and body region: the genera *Actinotalea* and *Paracoccus* were the most common in teeth, whereas *Dermacoccaceae* was the most common in feet. Furthermore, there were differences by relative quantity of cortical bone, so there appear to be influences on microbial communities by bone microstructure based on the available void space or nutritive differences. *Clostridium*, a well-known collagenase producer present in the human gut, was associated with a decrease in human DNA concentration, which might explain why the bones located near the gut provide the lowest DNA quantities; however, this hypothesis needs to be explored further with absolute abundance measurements and a larger sample [51].

Future trends in this field should include two perspectives. On the one hand, studies should be performed to assess changes in the soil microbiome during skeletal remains decomposition, not only near the bones but with various distances between the soil sample taken and the human remains. On the other hand, the microbiome found might correlate with the conservation of the skeletal remains’ DNA; thus, the success of DNA typing could be assessed by the soil microbiome. This correlation appears likely considering that, for instance, high humidity and an acid pH value predict low short tandem repeat typing success. Furthermore, those conditions would lead to the development of certain microorganisms whose appearance would alert researchers.

## 3. Challenges and Limitations

Microbiome forensics appears to be a highly promising field in forensic science, but there are still some hurdles to be overcome to be accepted as evidence in court, which is a primary goal of forensic scientists. They seek to prove that a person is or is not involved in a criminal event. Daubert v. Merrell Dow Pharmaceuticals (1993) subsequently laid these foundations in North American law and international laws regarding how science should be presented in court. More precisely, there are criteria for any science to be presented in court as evidence, including whether the technique has been tested in field conditions, whether it has been subjected to peer review, whether the rate of error is known, standardisation and whether it has been generally accepted in the scientific community [56]. In addition, the calculation of the likelihood ratio, recommended as a best practice by the European Network of Forensic Science Institutes [57], is not currently available for microbiome forensics. 

The microbiome might be an excellent predictor of geolocation, allowing researchers to connect a person to a certain place; however, there are issues to be addressed. Some of the elements to be considered are summarised in Table 4.

To address these limitations, three recommendations can be made to ensure the future of microbiome forensic research [28]:

(1) *Standardisation of Sample Protocols*. Collection, storage, analysis and interpretation of forensic microbiome samples need a standard protocol accepted by the scientific community so the results achieved become reproducible and repeatable.

(2) *Creation of a Robust and Reliable Microbiome Database*. Databases are one of the cornerstones of the forensic sciences, given that they can obtain metadata associated with certain microbiota (geographic origin, ethnic group, etc.) and allow for statistical treatment of the results with the calculation of a likelihood ratio. 

(3) *Performance of Studies with Many More Samples*. There is still little research on the various aspects of the microbiome. More research with a larger number of samples needs to be conducted in order to achieve representative results.

Specific issues also address microbiome geolocation (see Table 5), especially regarding variations in the soil and surface microbiome due to seasonal changes or climatological variations, as well as abiotic changes related to soil composition fluctuations and biotic changes due to the proliferation of decomposing bacteria during body decay. Soil sample conservation procedures, local sampling or soil microbiome monitoring are possible solutions to these challenges; however, more research is needed to develop and validate these possibilities.

To address these limitations, a database on the available microbiome datasets was built: the Forensic Microbiome Database, FMD (http://fmd.jcvi.org/about.php). It is composed of approximately 20,000 human 16S rRNA NGS samples from several body sites, capturing the metadata of geolocation, healthy/non-healthy status and other variables. The website allows researchers to compare microbiomes from various locations and body sites with the aim of predicting the geolocation of a given sample [64]. Although most of the data are from the United States, it is a promising tool for worldwide microbiome researchers.

## 4. Conclusions

Massive parallel sequencing technologies have led to the development of microbiome and microbiota studies due to the ability to distinguish between species by 16S rRNA sequencing. Since 2001, there has been increasing interest in microbiology in the forensic science community, especially regarding its applications to bioterrorism investigation. However, the use of forensic microbiomics has expanded in three main fields: identification, post-mortem interval establishment and geolocation. Forensic microbiome geolocation has been well explored in the literature, leading to several potential applications, such as city planning, city geolocation, soil evidence geolocation, room inhabitant determination, home cohabiting establishment, altitude and latitude estimation and even human trafficking in humanitarian programs such as DNA-Prokids or ancient DNA studies.

However, this potential tool needs to address several issues if its aims are to be achieved in the scientific community and in court. Forensic microbiome studies in general still face challenges, such as microbiome transfer, sample collection, DNA extraction, sequencing and analysis and training and data interpretation. In particular, geolocation as an application of forensic microbiomics presents specific difficulties: temporal mismatch, post-mortem soil microbiome alterations, abiotic soil variables affecting the microbiome and in vivo microbiome bias and lifestyle factors that affect the results and therefore the conclusions. 

To address these difficulties, three main recommendations can be made. First, there is a need for standardisation of the microbiome DNA analysis techniques to make comparisons between studies and arrive at comparable conclusions. Second, strong microbiome databases built from research data are needed if geolocation is to be implemented, to increase the accuracy of the results obtained and to provide a likelihood ratio of that result. Third, and no less important, further research is needed with a larger number of samples, so that the current applications and limitations of this promising technique can be assessed.

## Figures and Tables

**Table 1:** Sampling protocol from Earth Microbiome Project [40].

Sampling	Samples Should Be Collected Fresh and Then Frozen without Using Any Buffer or Solution.	
	Soil	Swabs
Procedure	Split fresh sample into 2 mL tubes (10) with, at least, 200 mg biomass and store at −80 or −20 °C.	Take 10 replicate swabs with no buffers or solutions and store in −80 or −20 °C
Shipping	Samples should be shipped with dry ice in an extruded polystyrene foam container or similar.	

**Table 2:** Commercial kits for microbiome DNA extraction.

Commercial Kit	Principle	Format	Time	Automation
*MagMAX Microbiome Ultra Nucleic Acid Isolation Kit (ThermoFisher Scientific)* [44]	Magnetic beads	100 reactions	~60 min	KingFisher^™^ Duo Prime, Flex and Presto
*Invitrogen PureLink Microbiome DNA Purification Kit (ThermoFisher Scientific)* [45]	Spin column	100 reactions	120 min	-
*QIAamp DNA Microbiome Kit (QIAGEN)* [46]	Silica columns	50 reactions	~180 min	-
*MO BIO’s PowerMag^®^ Soil DNA Isolation Kit (QIAGEN)* [47]	Magnetic beads	4 × 96 or 32 × 12	60–120 min	epMotion^®^

**Table 3:** Current applications of Forensic microbiome geolocation.

Forensic microbiome geolocation applications	City planning, public health and architectural design [17].
	City geolocation [18].
	Soil evidence geolocation [21].
	Room inhabitant determination and home cohabiting establishment [25].
	Altitude estimation [27].
	Latitude assessment [33].
	Human trafficking [12].
	Ancient DNA studies [51].

**Table 4:** Challenges in microbiome forensics [58].

Challenge	Considerations
Microbiome Transfer	- The human microbiome can be transferred between cohabitants, pets or unknown people by physical interaction between them. - The human microbiome can be deposited into built environments. - The persistence of the microbiome on various surfaces is not well studied.
Sample collection	- Forensic examiners, protective clothing or tools can introduce a foreign microbiome. - Evidentiary items have the potential to transfer the microbiome to forensic examiners or the laboratory. - Environmental changes affect the evidence microbiome, which complicates sample storage. - Laboratory background microbial DNA needs to be continuously monitored.
DNA extraction	- Difficulties in reproducing a sample profile. - Extraction kits contain a background microbiome (kitome). - Samples can be outcompeted by contaminating microbial DNA.
Sequencing and analysis	- Microbial contamination can take place during sequencing. - Lowtemplate microbial DNA samples. - Indexhopping (reads assign to the wrong sample) and batch effects (unwanted variations introduced by confounding unrelated factors). - Bioinformatics are constantly evolving and cases must be revised with the new information.
Training and interpretation	- Methods and protocols are not validated. - Proficiency tests need to be developed. - There are no established forensic databases. - Likelihood ratio (LR) calculation needs development. - Mixture of microbiome profiles. - Bioinformatics tools’ complexity.

**Table 5:** Forensic geolocation by microbiome analysis challenges and possible solutions.

Challenges		Possible Solutions
Temporal mismatch [59]	Significant differences in bacterial communities can be observed in the same soil sample if it is analysed at different times due to natural (seasonal) or artificial (storage) changes.	Soil sample enrichment so bacterial communities survive for longer periods.
Type of environment [60]	Water availability, changes in plant cover, input of fresh organic matter and temperature variations affect microbiota composition, so ecosystems with high variations in these factors can be challenging to analyse.	Sampling at local scales.
Post-mortem microbial communities [61]	Decaying body-associated microbiota changes the soil’s original bacterial communities; in addition, it changes during the various stages of decomposition, and there appear to be seasonal variations in the same soil.	Winter and summer characterization of the soil microbiome.
Abiotic soil variables [62]	pH and NH_4_^+^ fluctuations, as well as interactions between plants and microorganisms, affect soil bacterial communities.	Monitoring of soil microbiome changes.
In vivo microbiome bias [63]	In vivo microbiota can be a consequence not of the geographic place, but of certain sociodemographic aspects linked to culture or inequalities.	Continuous remapping.
In vivo microbiota lifestyle [64]	Among individuals in the same area with different lifestyles, diets or routines, the microbiome can vary. An individual microbiome changes drastically due to travelling, dietary changes or a recent infectious disease.	Additional studies on how infections affect host microbiota.

## Data Availability

Not applicable.
